# Botanical memory: five centuries of floristic changes revealed by a Renaissance herbarium (Ulisse Aldrovandi, 1551–1586)

**DOI:** 10.1098/rsos.230866

**Published:** 2023-11-08

**Authors:** Fabrizio Buldrini, Alessandro Alessandrini, Umberto Mossetti, Enrico Muzzi, Giovanna Pezzi, Adriano Soldano, Juri Nascimbene

**Affiliations:** ^1^ BIOME Lab - Dipartimento di Scienze Biologiche, Geologiche e Ambientali, Università di Bologna, Via Irnerio 42, 40126 Bologna, Italy; ^2^ Sistema Museale di Ateneo, Università di Bologna, Via Irnerio 42, 40126 Bologna, Italy; ^3^ Independent Researcher, Via G. Pilati 19, 40018 San Pietro in Casale (BO), Italy; ^4^ Dipartimento di Scienze e Tecnologie Agrarie e Alimentari, Università di Bologna, Viale G. Fanin 44, 40127 Bologna, Italy; ^5^ Independent Researcher, Largo Brigata Cagliari 6, 13100 Vercelli, Italy

**Keywords:** Ulisse Aldrovandi, alien species, Bologna, flora, global change, native species

## Abstract

We analysed the spatially explicit floristic information available in the herbarium of Ulisse Aldrovandi (1551–1586) to track floristic changes in the surroundings of Bologna across five centuries. Aldrovandi's data were compared with the *Flora della Provincia di Bologna* by Girolamo Cocconi (1883) and the Floristic Database of Emilia-Romagna (1965–2021). We explored potential variations in native range and life forms composition, and habitat affinity of the species in the three floras, also contrasting between native and alien species. Native species, mainly in terms of variations of hydro-hygrophytes, chamaephytes and therophytes, provide clear signals of human disturbance and habitat loss. Signals of climate change are provided by the high-mountain species, that were comparably rare between Aldrovandi and current flora and more represented in Cocconi, probably reflecting the effect of the Little Ice Age. Our findings also indicate the increasing importance of alien species from the Renaissance onwards. In this perspective, Aldrovandi's herbarium preserves the memory of the first signs of a radical transformation of the European flora and habitats. Finally, the study warns about the risk of dismissing herbaria and herbarium specimens collection, which would cause irreparable lacunas in our botanical memory, hindering our ability to predict biodiversity trajectories.

## 1. Introduction

Herbaria are experiencing a renewed interest for their historical importance, their usefulness in diachronical analyses of biodiversity (e.g. [1–3]), and new applications related to scientific and technological progress [4,5], such as the use of historical DNA in evolutionary studies [6] or tracking colonization pathways of invasive species [7]. In particular, the few Renaissance herbaria are fundamental for understanding the development of Botany as a science [8–10], as well as for evaluating environmental changes across a multiple-century timespan.

Renaissance herbaria allow to interpret the polynomial nomenclature, delineate the exchange networks among botanists [11], track the history of the cultivation of edible species, the introduction of exotic plants [12–14] and past uses of certain species [15]. The original purpose of these *horti sicci* (i.e. dried plant gardens) was to discuss the identity of plant species, which were compared with the descriptions provided in the ancient treaties by Pliny the Elder, Dioscorides and Galenus, to understand which were the right species to be used for pharmaceutical purposes (e.g. [11,16]).

However, for today's science, one of the most interesting uses of Renaissance herbaria is unravelling their botanical memory to track the effect of global changes on biodiversity. In this perspective, their importance is related to the availability of information on the collection period and sites. While the collection period is usually available, or can be somehow inferred from complementary documents (e.g. correspondence exchanges), collection sites are almost always missing or mentioned in very vague terms (i.e. referred to a region or a country), thus hampering explicit spatial analyses through retrospective georeferencing (*sensu* [17]) to detect, quantify and interpret spatial floristic changes across time slices.

In this context, the herbarium of Ulisse Aldrovandi (Bologna, 1522–1605) is an outstanding exception. It was prepared between 1551 and 1586, and currently it is preserved and digitized at the herbarium of the University of Bologna (BOLO). It includes about 5000 specimens, many of them collected in the surroundings of Bologna (northern Italy), for which quite accurate information on the collection site is available. Actually, this information is not directly reported among the writings near the specimens (pre-Linnaean herbaria never bear labels on the sheets), but can be retrieved from manuscripts associated with the herbarium. To our best knowledge, this is the most ancient case at the global level of herbarium samples for which collection places are known, that allows to use specimens collected half a thousand years ago like those collected nowadays to track floristic changes across a time period of about 500 years. The uniqueness of this botanical memory is further enhanced by the approach to collection adopted by Aldrovandi that, at least for the surroundings of Bologna, was intended to maximize the floristic survey of the territory. This situation, coupled with his ability to identify species on the basis of even subtle morphological characters [18,19], increases the possibility of comparisons with more recent collections in the same area.

The province of Bologna is a floristically well-studied region that, thanks to the botanical tradition that started since the Renaissance, was explored by many botanists across five centuries. The initiator of this tradition was Luca Ghini, a personality of European fame in the field of natural sciences, who founded a true school of Botany in the 1530s, when he was professor in Bologna (e.g. [20,21]). In those decades Bologna became a sort of cradle of modern Botany and herbaria, and various important collections originated in this area and time period: apart from Aldrovandi's one, we remember for example the herbarium kept in Rome in Biblioteca Angelica and the *En Tibi* herbarium, both by Francesco Petrollini [22–24]. In particular, 300 years after Aldrovandi, several surveys were carried out by Antonio and Giuseppe Bertoloni (1820s–1870s) and their collaborators, contributing to an exhaustive flora of the Bologna province, published by Girolamo Cocconi in 1883 [25,26]. In the last decades (i.e. since the 1970s), additional research was focused on this area in the context of a floristic mapping project for the entire region (Emilia-Romagna), resulting in a floristic database that currently includes more than 500 000 records [25].

In this study, we first analysed the flora of the whole province of Bologna during the Renaissance on the basis of the herbarium of Aldrovandi, then we tracked floristic changes across a timespan of five centuries, comparing three floristic datasets retrieved from: (i) the herbarium of Aldrovandi, (ii) the Flora by Cocconi, and (iii) the floristic database of Emilia-Romagna, extracting records referring to the last six decades (1965–2021). The comparison refers only to the plain area, the best explored part of the Bologna province, where floristic exploration was consistently comparable across the three datasets. In particular, we explored potential variations in native range and life forms composition, and habitat affinity of the species in the three floras, also contrasting between native and alien species. Floristic patterns were then interpreted in the light of global changes, thus considering increased human development, land use change, alien flora invasions and climate change.

## 2. Material and methods

### 2.1. The herbarium of Ulisse Aldrovandi

**Figure 1.**
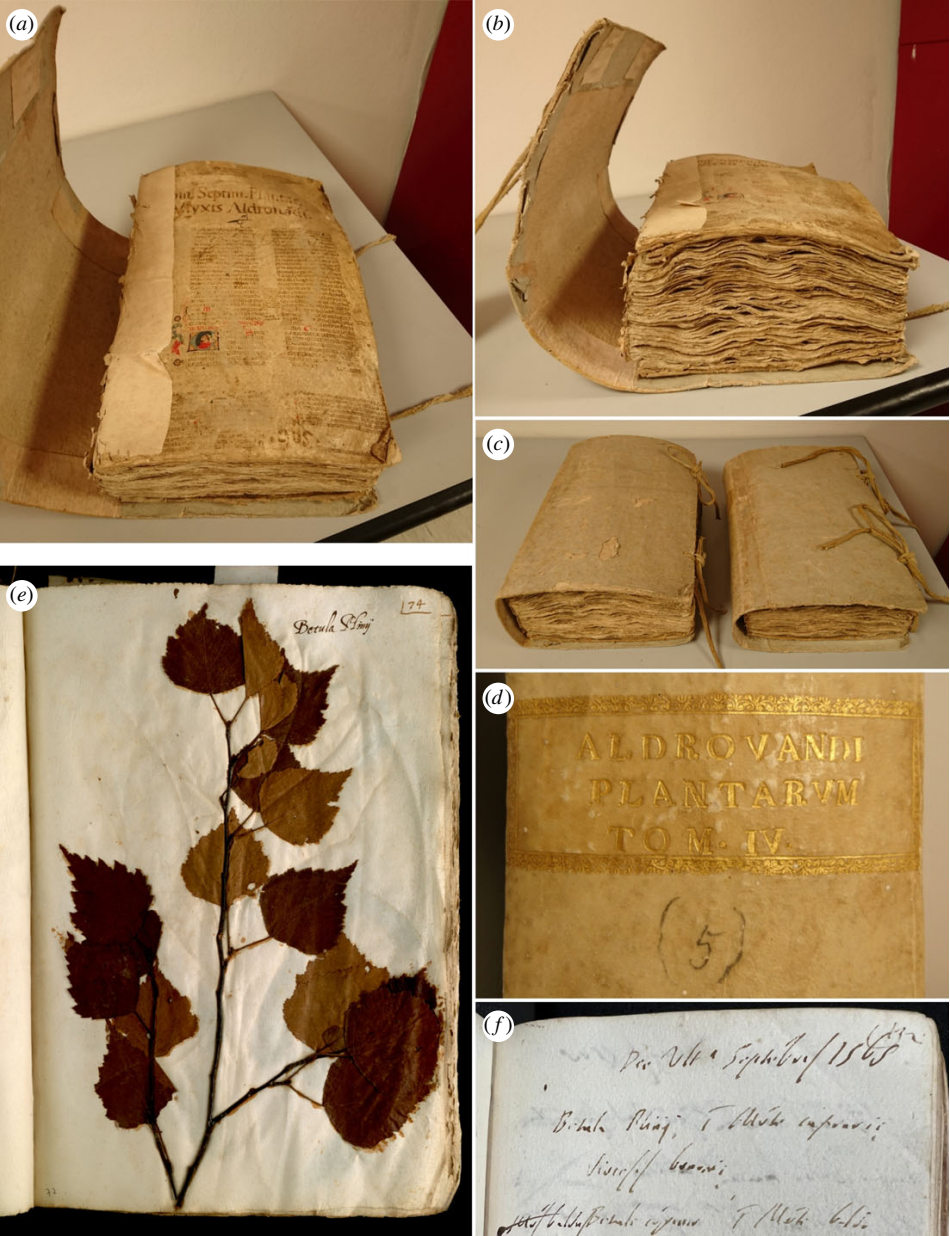
The herbarium of Ulisse Aldrovandi. (*a*) view of one of the volumes (on the inner cover *Tomum Septimum Plantarum Ulyxis Aldrovandi* is written), (*b*) another view of the same volume, (*c*) view of volumes V and VII, with the external covers visible, (*d*) view of the spine of volume V, (*e*) view of one of the samples collected in the province of Bologna: vol. I, c. 74r.—*Betula Plinij* (= *Betula pendula* Roth.), (*f*) manuscript page bearing the indication of the collection place of the sample: Biblioteca Universitaria di Bologna, Fondo Ulisse Aldrovandi, ms. 136-III c. 132r., *Betula Plinij in Monte caprario diocesis bononiæ* (= on Mt. Capra in the diocesis of Bologna). Photographs: (*a*–*d*) F. Buldrini, (*e*) © Alma Mater Studiorum Università di Bologna—Biblioteca Universitaria di Bologna (reproduction and duplication forbidden), (*f*) N. Di Tommaso (Dipartimento di Filosofia e Comunicazione, Università di Bologna).

The herbarium of U. Aldrovandi, preserved in the *Herbarium Universitatis Bononiensis* (BOLO), consists of 15 books (*ca* 34 × 24 cm in length and width), which were prepared between 1551 and 1586 ([19,27]; figure 1). Each of them includes from 169 to 581 specimens glued to the sheets, for a total of more than 5000 specimens originally: at that time, it was the richest collection of dried plants existing in Europe [27]. During the centuries, however, 108 sheets were removed from the volumes and incorporated in other herbaria, or simply dispersed [28,29], therefore, the specimens are currently 4841.

From a historical and scientific perspective, the importance of this herbarium is inestimable [8,9,30]. On a European scale, it contains the oldest specimens of some species currently used for food, ornament or medicinal purposes [31–34] that were introduced from the New World (e.g. *Mirabilis jalapa* L., *Nicotiana tabacum* L., *Opuntia ficus-indica* (L.) Mill., *Solanum lycopersicum* L.), from Africa, or central and eastern Asia (e.g. *Aloe vera* (L.) Burm. fil., *Canna indica* L., *Diospyros lotus* L., *Melia azedarach* L., *Nardostachys jatamansi* (D. Don) DC.). In addition, which is even more important, collection sites of the *exsiccata* are documented. In various cases, they are indicated in a rather generic way, whereas sometimes the precision is comparable to current standards. At times, the collection site is complemented by observations on species frequency and abundance, ecology, local names or uses in folk medicine.

### 2.2. Study area

**Figure 2.**
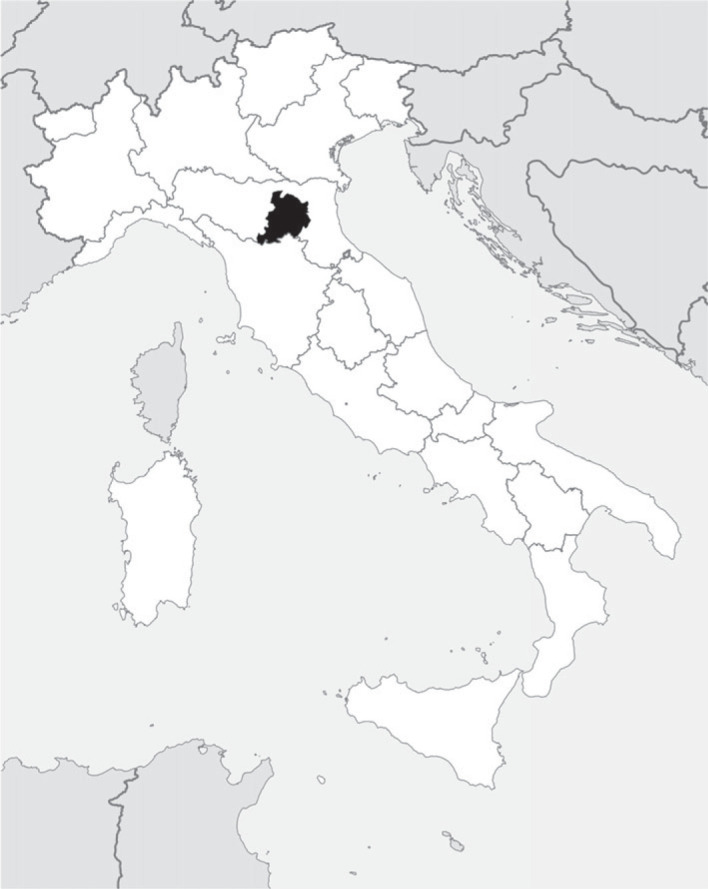
Location of the study area within the Italian territory.

The territory explored by Aldrovandi roughly corresponds to the province of Bologna (Emilia-Romagna, northern Italy; figure 2), spanning an altitudinal range between sea level and 1944 m.a.s.l. (Corno alle Scale summit). The plain part of the province is an alluvial area originated by the River Po and its tributaries, with river sediments and calcareous and argillaceous soils. In the sixteenth century, large areas of this plain were occupied by a wetland *continuum* called Valle Padusa, a wide system of more or less deep freshwater marshes, partially forested [35], which was progressively reclaimed for agriculture [36–38]. In the remnant parts of the plain, the landscape was dominated by agricultural areas and residual forests [39]. The potential natural vegetation of the plain area would be a mixed deciduous forest characterized by *Quercus robur* L., *Carpinus betulus* L. and *Fraxinus angustifolia* Vahl, substituted by riparian forests along rivers and water basins and herbaceous formations of hydro-hygrophytes in wetland areas.

The hilly–mountainous part of the province is characterized by moderate altitudes not exceeding 600–700 m.a.s.l.; only in proximity of the Apennine watershed higher altitudes are reached (from 1200 up to 1944 m.a.s.l.). Geological and pedological characters are extremely heterogeneous. The substrate is formed by marl, sandstone, clays and chaotic complexes of various rocks immersed in an argillaceous matrix, and large gypsum outcrops [40]. The potential natural vegetation of the hilly–mountainous part would be a deciduous forest dominated by *Quercus pubescens* Willd., *Q. petraea* (Matt.) Liebl. or *Q. cerris* L. up to *ca* 900–1000 m.a.s.l., then *Fagus sylvatica* L. forests in the montane belt, up to 1700 m.a.s.l.; in the highest part there are sub-alpine grasslands and heathlands dominated by *Vaccinium* spp. [25].

### 2.3. Data extraction from the herbarium of Aldrovandi

The herbarium was studied by one of us (A.S.) between the late 1990s and early 2000s (the collection, entirely digitized, is available at http://137.204.21.141/aldrovandi/Explore), with identification of the *exsiccata*, correction of former identifications and, whenever possible, attribution of the collection site as retrieved from Aldrovandi's manuscripts [18,19,27,41–43]. For this study, we extracted only the 1899 specimens referring to the province of Bologna. Due to the poor state of preservation, 142 of them were not identifiable or were identified only at the genus level; therefore, they were discarded from the analyses. The remnant 1757 specimens were checked in order to separate (and therefore treat as alien):

(1) exotic species occurring only in cultivation in private gardens or in the Botanical Garden of Bologna, which generally do not tend to naturalize;
(2) species cultivated for food, dyeing or pharmaceutical uses, which are not native to the explored area and do not tend to naturalize; of this category we maintained among the native species only those that could be present as casual, since they may still be sporadically observed throughout the territory;
(3) species whose presence in the study area is improbable for geographical or climatic reasons, based on today's knowledge of the territory and its flora and even in light of the environmental variations that occurred throughout the centuries (e.g. species which are currently present only in the Alps or in environments with a strictly Mediterranean climate).

Scientific names were updated following Pignatti *et al*. [44]; wherever necessary, species identification was corrected according to current taxonomic interpretation. For each species, we retrieved life form and native range from Pignatti *et al*. [44], grouping distributional ranges on the basis of consolidated criteria [45–48]. Alien species were divided into archaeophytes, neophytes and cryptogenic, and current status in Italy (casual, naturalized, invasive) was added, following the Portal to the Flora of Italy [49]. To analyse species loss in Aldrovandi's and Cocconi's floras, protection and extinction level of the species were retrieved from Rossi *et al*. [50,51], Regione Emilia-Romagna [52], Pezzi *et al*. [25], Portal to the Flora of Italy [49]. The species were then categorized into ecological groups based on their habitat affinity, according to Pignatti *et al*. [44]:

(1) grasslands (species of open and sunny places),
(2) forests (nemoral species, or mostly typical of shady places),
(3) mountains (species of the suprasylvatic belt),
(4) wetlands (hygrophilous and palustrine species),
(5) shallow waters (aquatic species),
(6) ruderal (ruderal, synanthropic and segetal species),
(7) rocks (rupicolous species, or mostly typical of substrates made of debris or rubble),
(8) euryoecious (species with a broad ecology, but not ascribable to the ruderal category).

### 2.4. Data analysis

To have an overview of the flora of the Bologna province deducted from the Erbario Aldrovandi, this flora was analysed in life forms, native ranges and species currently protected or extinct in the study area.

To track changes in the flora of the Bologna province across five centuries, we compared the data extracted from Aldrovandi's herbarium with those available in the two most important floristic sources for this area:

(1) the *Flora della Provincia di Bologna* by Cocconi [53], that was entirely digitized and georeferenced [26],
(2) the Floristic Database of Emilia-Romagna (online at https://bbcc.ibc.regione.emilia-romagna.it/flora-emilia-romagna/), maintained and updated by one of us (A.A.), that includes all floristic data available for this region.

The comparison was performed examining the plain area, since it is the most highly explored part of the province across the three periods, thus allowing more consistent comparison among them; moreover, this area is geomorphologically homogeneous and can clearly be marked off.

To extract the species list of the plain area from Aldrovandi's herbarium, we first considered all the spontaneous species collected in the so-called *agro bolognese* (the territory around the city of Bologna) and in the plain localities. Then we eliminated all the species that surely could not be present in the plain, according to the following criteria:

(1) chorology (orophytes, arctic-alpine, endemic to Italy—the latter often are Apenninic or in any case mountain species),
(2) life form (chamaephytes, that were probably impossible to find in Aldrovandi's time, the plain of Bologna being dominated by large marshes and swamps),
(3) ecology (rupicolous or petrophilous species, such as many *Dianthus* and *Teucrium*, for the lack of these habitats in the plain).

To extract the species list from Cocconi's flora, we filtered the dataset retrieving only the records from localities of the plain area.

To extract the species list from current data, we filtered the records of the Floristic Database of Emilia-Romagna, maintaining only those referred to the plain localities collected between 1965 and 2021 (electronic supplementary material, S1). The threshold of 1965 is related to the transition from the traditional to modern mechanized agriculture [48] and to the first modern studies on the flora of the province of Bologna that date to that period [25].

To avoid forcing the comparison, we considered at the group level certain species traditionally difficult to separate into those currently recognized ([44,54]; table 1).

**Table 1.** Species treated at the group level, since the separation from other congeneric species has long been difficult and controversial until recent times.

group of species	taxa included
*Achillea* gr. *millefolium*	*A. collina* (Wirtg.) Heimerl, *A. millefolium* L., *A. roseoalba* Ehrend.
*Alchemilla* gr. *alpina* and *Alchemilla* gr. *vulgaris*	all species of this genus were reduced to the two traditional groups, since most records—especially ancient ones—are practically always reported in this form
*Avena* spp.	*A. barbata* Pott ex Link, *A. fatua* L., *A. sativa* L., *A. sterilis* L.
*Callitriche* spp.	*C. brutia* Petagna subsp. *hamulata* (W.D.J. Kock) Bonnier et Layens, *C. cophocarpa* Sendtn., *C. platycarpa* Kütz., *C. stagnalis* Scop.
*Leucanthemum* gr. *vulgare*	*L. pallens* (Gay ex Perreym.) DC., *L. vulgare* Lam.
*Pulmonaria* gr. *officinalis*	*P. apennina* Cristof. et Puppi, *P. hirta* L., *P. officinalis* L.
*Rosa* gr. *canina*	*R. canina* L., *R. corymbifera* Borkh., *R. rubiginosa* L.
*Rubus* gr. *ulmifolius*	*R.* sect. *Corylifolii* Lindl., *R.* sect. *Rubus* L. subsect. *Hiemales* E.H.L. Krause ser. *Discolores* (P.J. Müll.) Focke
*Stellaria* gr. *media*	*S. media* (L.) Vill., *S. neglecta* Weihe, *S. pallida* (Dumort.) Piré, *S. ruderalis* M. Lepší, P. Lepší, Z. Kaplan et P. Koutecký
*Vicia sativa*	*V. sativa* L. *sensu lato*, here included all the subspecies

The three datasets were analysed and compared on the basis of life forms and native ranges. In R v. 4.2.2 [55], the following statistical analyses were performed, on the frequency data of the tables prepared:

(1) a correspondence analysis (*FactoMineR* and *factoextra* packages), to associate the datasets (3) with life forms (7) and native ranges (7);
(2) a log-linear model analysis (*Crosstabs.Loglinear* package) to test the differences between the principal effects (datasets, life forms and native ranges) and their interaction;
(3) once the statistical significance was demonstrated, a reassembling of the contingency tables (*sensu* Sigiel & Castellan [56]) and a clustering *sensu* Greenacre [57] were performed to understand how the statistical differences highlighted among the three datasets were reciprocally characterized.

The three datasets were also compared in pairs by Jaccard's diversity index.

Finally, we calculated the extinction rate on the floristic lists recorded by Aldrovandi and Cocconi for the entire province of Bologna; then, to test the differences in the percentage of local extinction among the diverse habitats and between periods, we performed a *χ*² test and a clustering *sensu* Greenacre [57], taking into account the species recorded by Aldrovandi and Cocconi that are either extinct today or not confirmed for at least the past 40 years.

## 3. Results

### 3.1. Outline of Aldrovandi's flora

Based on the 1757 identifiable specimens of his herbarium, Aldrovandi's flora of the province of Bologna consisted of 980 currently recognized species (electronic supplementary material, S2), including 819 native (1482 specimens) and 161 alien to this territory (275 specimens); 11 out of 161 species are alien at the national level.

#### 3.1.1. Native species

The species currently extinct or unknown at the regional scale are 13, those extinct in the province of Bologna are 8 and those needing to be confirmed in the region are 8. Among these species, 11 were hydro-hygrophilous (e.g. *Berula erecta* (Huds.) Coville, *Hippuris vulgaris* L., *Ranunculus lingua* L.). The species protected at a regional or international level are 30 (i.e. 3.6%): 20 are orchids, typical of open and sunny places (e.g. *Anacamptis morio* (L.) R.M. Bateman, Pridgeon et M.W. Chase, *Himantoglossum adriaticum* H. Baumann, *Spiranthes spiralis* (L.) Chevall.) or forest habitats (e.g. *Epipactis helleborine* (L.) Crantz, *Listera ovata* (L.) R. Br., *Platanthera bifolia* (L.) Rich.). The others are mainly species of hilly or mountain forests (e.g. *Gentiana asclepiadea* L., *Lilium martagon* L.), or wetlands (e.g. *Leucojum aestivum* L., *Nymphaea alba* L.). Therophytes are 26.1% of the species (table 2), whereas hydrophytes and helophytes are marginal (less than 4%). The Eurasian and Mediterranean species together account for 66.4% of the total, followed by microthermal (18.3%) and cosmopolitan species (13.1%; table 3).

**Table 2.** Biological spectrum for the species registered by Ulisse Aldrovandi (sixteenth century) in the territory studied.

life form	value (%)	
	native	alien
chamaephytes	4.0	10.6
geophytes	14.9	11.8
helophytes	0.9	1.2
hemicryptophytes	40.8	18.0
hydrophytes	2.8	—
phanerophytes	10.4	28.0
therophytes	26.1	30.4

**Table 3.** Chorological spectrum for the species registered by Ulisse Aldrovandi (sixteenth century) in the territory studied.

native range	value (%)	
	native	alien
Eurasian species	40.8	23.0
Mediterranean species	25.6	37.3
boreal and montane species	18.3	11.8
cosmopolitan species	13.1	1.9
species from the tropical and intertropical regions of the Old World	1.0	12.5
species existing only in cultivation, in the reference territory	0.5	6.8
species coming from the Americas	—	4.4

#### 3.1.2. Alien species

The species currently extinct or unknown on a regional level are 64 (12 of them have not been reported in Italy at all), those extinct in the province of Bologna are 6 and those needing to be confirmed in the region are 6. Most of them were cultivated as ornamental (e.g. *Cistus* spp., *Hemerocallis lilioasphodelus* L., *Platanus orientalis* L.) or medicinal species (e.g. *Citrus medica* L., *Leontice leontopetalum* L., *Mandragora officinarum* L.). Three species (i.e. 1.9%) are protected on a regional or international scale (CITES): *Cyclamen purpurascens* Mill., an Alpine species, *C. repandum* Sm. and *Vinca major* L., typical of Mediterranean Italy. Neophytes are 19, archaeophytes 43; the species native to Italy, but alien to the province of Bologna, are 80. Therophytes dominate (30.4%), followed by phanerophytes (28.0%; table 2). The Mediterranean and Eurasian species prevail (60.3%), followed by tropical and microthermal species (12.5% and 11.8%, respectively); some minor groups sum up to 9.4% (table 3).

Additional information on native and alien species is reported in electronic supplementary material, S3 and S4, respectively.

#### 3.1.3. Geographical distribution of Aldrovandi's floristic records

**Figure 3.**
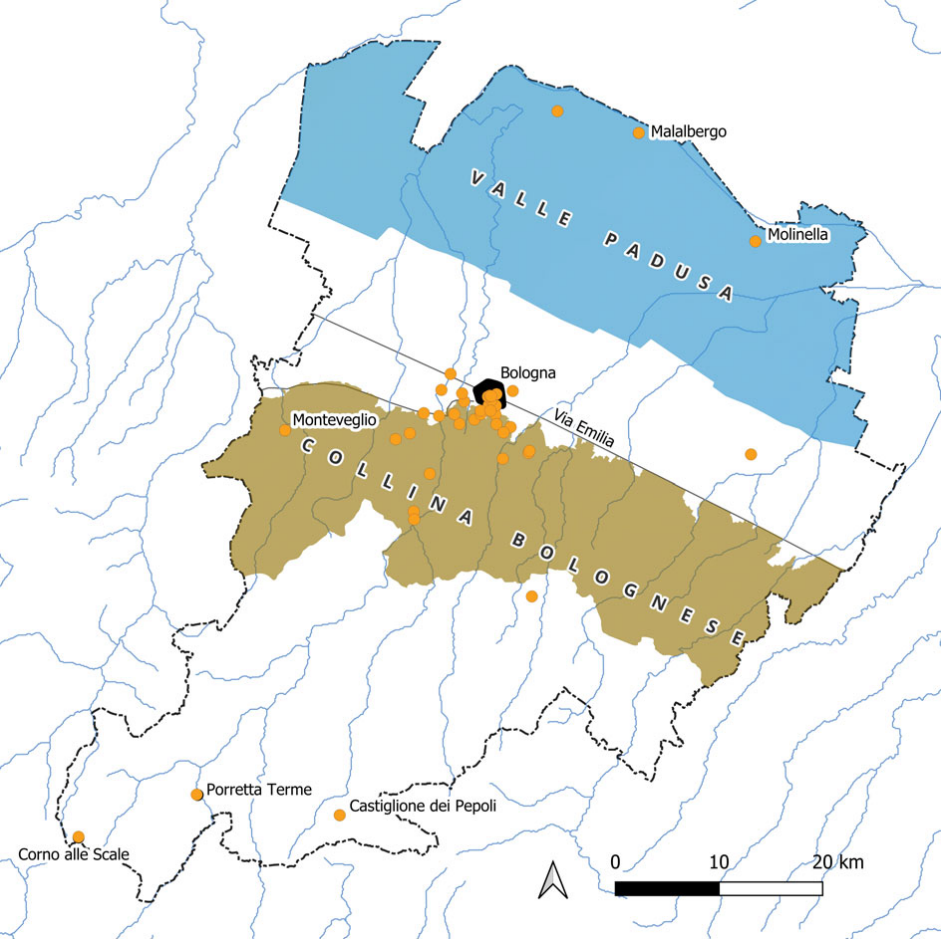
Map of the localities cited by Ulisse Aldrovandi as collection sites for his exploration of the territory of Bologna.

The species were collected in 46 sites, of which 42 are referred to a precise toponym; the remaining ones are quite vaguely attributed to Bologna's territory (figure 3). Among the 42 toponyms, 27 are included in a 10 km radius from Bologna city centre and are located east, south and west of the urban area. Other sites of particular exploration are the mountain area of the Corno alle Scale and the large marshes of the Valle Padusa, at about 60 km southwest and 30 km north-northeast from Bologna, respectively. The localities with most species are the *agro bolognese* (552 native and 50 alien species), the urban area of Bologna (181 native, 119 alien species), the Valle Padusa (46 native species), the mountains of Bologna (29 native, 1 alien species) and the Corno alle Scale massif (19 native species).

### 3.2. Comparison among Aldrovandi, Cocconi and current flora

In Aldrovandi, the species recorded in the plain area were 585 (8.5% alien), in Cocconi 310 (6.8% alien), and in current flora 1069 (21.6% alien).

**Figure 4.**
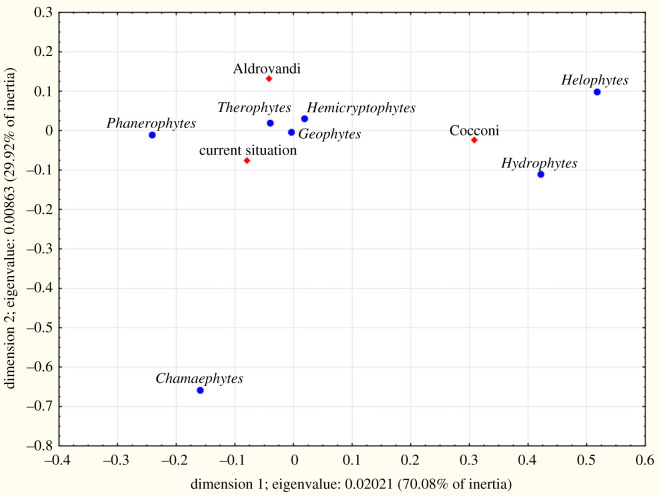
CA plot of the life forms for native species for the plain area of the territory studied: comparison among the species registered by Ulisse Aldrovandi (sixteenth century) and Girolamo Cocconi (nineteenth century) and those known today (1965–2021), reported in the Floristic Database of Emilia-Romagna.

**Figure 5.**
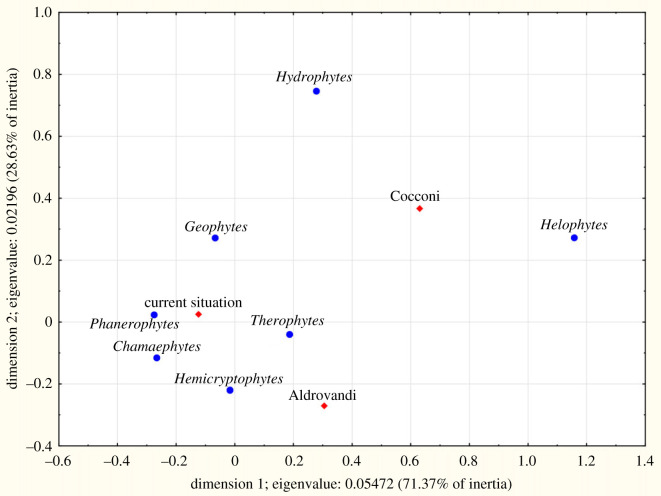
CA plot of the life forms for alien species for the plain area of the territory studied: comparison among the species registered by Ulisse Aldrovandi (sixteenth century) and Girolamo Cocconi (nineteenth century) and those known today (1965–2021), reported in the Floristic Database of Emilia-Romagna.

Among native species (table 4), the contribution of phanerophytes was almost the same between Aldrovandi and current flora (approx. 10%), whereas in Cocconi it was only 4.5%. Hydrophytes and helophytes in Cocconi were nearly thrice the value of the other two periods. Chamaephytes increased from Aldrovandi to current age. The correspondence analysis (CA) showed similarity between Aldrovandi and current data, whereas Cocconi was very different, especially considering helophytes and hydrophytes (figure 4). The differences among the three periods always were highly significant (*p* = 0.000 in the log-linear model). Among alien species (table 4, figure 5), in Cocconi the hydrophytes and helophytes together accounted for 9.6% of the list, about 2% in Aldrovandi and current flora. The latter period also showed a more abundant presence of phanerophytes. The differences among the three periods always were highly significant (*p* = 0.000 in the log-linear model).

**Table 4.** Biological spectrum for the plain area of the territory studied: comparison among the species registered by Ulisse Aldrovandi (sixteenth century) and Girolamo Cocconi (nineteenth century) and those known today (1965–2021), reported in the Floristic Database of Emilia-Romagna.

life form	value (%)					
	Aldrovandi		Cocconi		today	
	native	alien	native	alien	native	alien
chamaephytes	0.2	2.0	1.4	0.0	2.7	2.6
geophytes	12.5	4.0	12.5	14.3	12.6	10.5
helophytes	1.3	2.0	2.8	4.8	0.8	0.4
hemicryptophytes	39.8	16.0	39.6	4.8	36.9	11.4
hydrophytes	4.1	0.0	11.1	4.8	4.9	1.3
phanerophytes	9.9	18.0	4.5	9.5	10.7	34.9
therophytes	32.1	58.0	28.1	61.9	31.1	39.7

**Figure 6.**
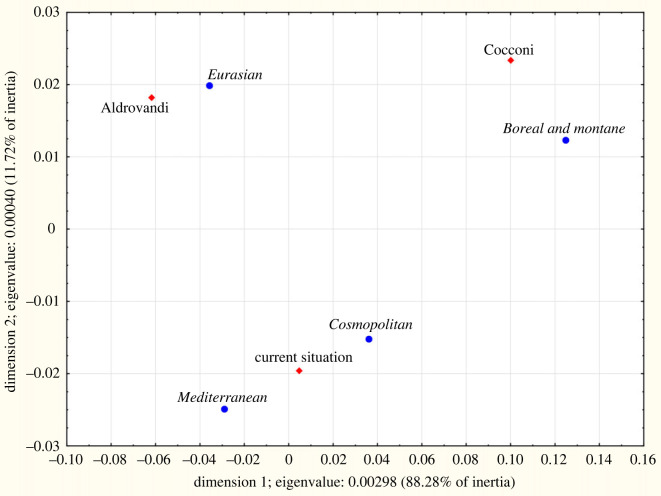
CA plot of the native ranges for native species for the plain area of the territory studied: comparison among the species registered by Ulisse Aldrovandi (sixteenth century) and Girolamo Cocconi (nineteenth century) and those known today (1965–2021), reported in the Floristic Database of Emilia-Romagna.

**Figure 7.**
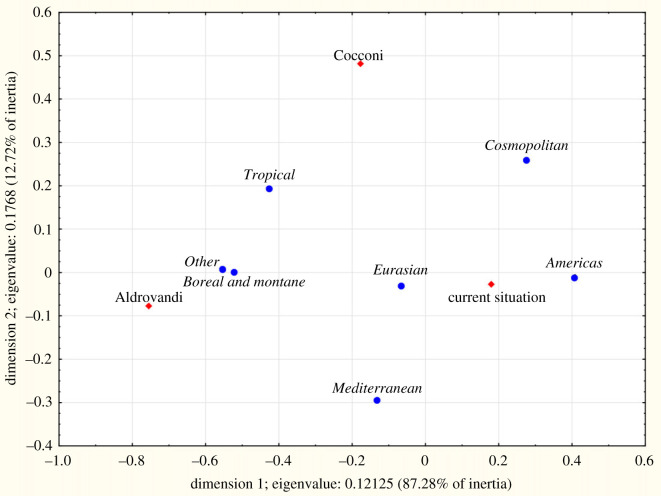
CA plot of the native ranges for alien species for the plain area of the territory studied: comparison among the species registered by Ulisse Aldrovandi (sixteenth century) and Girolamo Cocconi (nineteenth century) and those known today (1965–2021), reported in the Floristic Database of Emilia-Romagna.

Native ranges for autochthonous species (table 5) showed differences for microthermal (15.7% in Cocconi, 11–12% in Aldrovandi and current flora), Eurasian (42.6% in Aldrovandi, approx. 39% in Cocconi and modern ones) and Mediterranean species (25.2% in Cocconi's flora, more than 27% in Aldrovandi's and current flora). The differences among the three periods always were highly significant (*p* = 0.000 in the log-linear model); in particular, the correspondence analysis showed that Cocconi's flora was different from the others (figure 6). Among alien species (table 5, figure 7), in Aldrovandi those of the Old World were predominant (70.0%) while the Americans were 4.0%; in Cocconi Old World species were 47.7% and the American 23.8%; in current flora, these two groups accounted for 45.0% and 38.9%, respectively. The differences among the three periods always were highly significant (*p* = 0.000 in the log-linear model).

**Table 5.** Chorological spectrum for the plain area of the territory studied: comparison among the species registered by Ulisse Aldrovandi (sixteenth century) and Girolamo Cocconi (nineteenth century) and those known today (1965–2021), reported in the Floristic Database of Emilia-Romagna.

native range	value (%)					
	Aldrovandi		Cocconi		today	
	native	alien	native	alien	native	alien
Eurasian species	42.6	30.0	39.2	23.8	39.6	25.3
Mediterranean species	27.3	14.0	25.2	0.0	27.8	9.6
boreal and montane species	10.8	8.0	15.7	4.8	12.5	2.6
cosmopolitan species	17.4	2.0	19.6	14.3	18.9	8.7
species from the tropical and intertropical regions of the Old World	0.9	18.0	0.4	19.1	0.7	7.5
species coming from the Americas	—	4.0	—	23.8	—	38.9
other species	1.0	24.0	—	14.3	0.4	8.3

The three floras show a low similarity when compared by Jaccard's index: *J*_Aldrovandi−Cocconi_ = 0.19, *J*_Aldrovandi−current situation_ = 0.28, *J*_Cocconi−current situation_ = 0.19.

### 3.3. Extinction rate in Aldrovandi and Cocconi

The extinction rate for Aldrovandi was 2.96% (on a total of 980 species), for Cocconi 4.71% (on a total of 1699 species); the difference between these rates was statistically significant (*χ*^2^ = 4.87, *ν* = 1, *p* < 0.05). The two authors were reciprocally characterized as follows: in Aldrovandi the extinct species were mainly related to grasslands and ruderal environments; in Cocconi they were mainly hydro-hygrophilous (with a predominance of the palustrine and hygrophilous), followed by those of mountain areas (table 6). The differences in the extinction level among habitat types were significant: *χ*^2^ = 18.917, *ν* = 7, *p* < 0.01.

**Table 6.** Synthesis of the extinct and not confirmed species (i.e. last observed at least 40 years ago) in Aldrovandi's and Cocconi's floras of the territory studied.

species category	value (%)	
	Aldrovandi	Cocconi
forest species	0.0	11.3
grassland species	34.5	13.8
mountain species	3.4	11.3
rock outcrops species	0.0	6.3
ruderal species	20.7	7.5
shallow waters species	20.7	18.8
wetland species	17.2	31.3
euryoecious species	3.4	0.0

## 4. Discussion

The botanical memory preserved in Aldrovandi's herbarium allowed us to track floristic changes in connection with human disturbance, habitat loss and transformation, climate change and alien species invasion across 500 years. Ideally, our results contribute to shed light on the patterns of floristic changes that may have occurred in lowland temperate Europe, from the discovery of America up to the present day, paralleling the increase of human population. To have an idea, demographic trend in Europe (European part of the former USSR not included) shows an increase of 807% from 1500 to 2020; in the same time interval, Italian population experienced an increase of 564% [58,59].

The robustness of the use of the botanical memory included in Aldrovandi's herbarium is supported by the impressive number of species that he identified and collected, given that the concept of species was not developed as we understand it today, but was exclusively based on more or less strict morphological similarities [60,61]. Moreover, the floristic exploration in Europe was still at the very beginning, because the concept of flora was not yet defined scientifically (cf. [62,63]). In this perspective, Aldrovandi introduced a new paradigm in Renaissance botanical science, attempting for the first time to survey the biodiversity of a territory systematically [64–66]. This may explain the relatively high level of floristic exploration, albeit unbalanced in favour of the areas surrounding Bologna. To surpass the 1000 plant species for the Bologna province we have to wait for the nineteenth century, thanks to the joint work by Antonio and Giuseppe Bertoloni [53].

The patterns of native species, mainly in terms of variations of hydro-hygrophytes and chamaephytes, provide signals of human disturbance and habitat loss. The latter are typical of arid environments and their increase may reflect the effect of reclamation of the Valle Padusa for agriculture, resulting in sandy soils colonized by xerophilous species [67]. Hydro-hygrophytes are much more numerous in Cocconi than in Aldrovandi and current flora. This may indicate more disturbed conditions of freshwater habitats at Cocconi's time compared with Aldrovandi, due to increasing land reclamation [36,67]. Beside some species typical of undisturbed conditions (e.g. *Nymphaea alba* L. or *Hippuris vulgaris* L.), reclamation works enhanced the establishment of several species related to eutrophic or turbid waters, such as *Nymphoides peltata* (S.G. Gmel.) Kuntze, *Persicaria hydropiper* (L.) Delarbre, *Potamogeton pectinatus* L. and *P. perfoliatus* L. [44], recorded by Cocconi but not by Aldrovandi, and, in parallel, caused the extinction of less tolerant species like *Cladium mariscus* (L.) Pohl, *Stratiotes aloides* L. and *Thysselinum palustre* (L.) Hoffm. [44,68], recorded only by Aldrovandi. Currently, the low diversity and abundance of hydro-hygrophytes is mainly related to the heavy human impact and the spreading of invasive species in plain regions [69–71].

Similar dynamics are corroborated by the pattern of therophytes, that are more represented in Aldrovandi's and current floras than in Cocconi. During the Renaissance, this may have been mainly related to the widespread presence of marshes subjected to seasonal flooding–drying cycles [72]. The floristic composition of these habitats is in fact characterized by annual species, tolerating submersion during spring and aridity in summer [73]. This view is supported by the occurrence, in Aldrovandi's flora, of many species typical of muddy soils and temporarily flooded environments, such as *Bidens cernua* L., *Blackstonia perfoliata* (L.) Huds., *Juncus bufonius* L. and *Lythrum hyssopifolia* L. Currently, the abundance of therophytes is mainly associated with human impact (intensive agriculture, urban sprawl, infrastructure building, etc.), as indicated by the commonness of several species in the genera *Amaranthus*, *Crepis*, *Eragrostis*, *Erigeron*, *Setaria*, etc.

Signals of climate change are provided by the high-mountain species, which were comparably rare in Aldrovandi's and current floras and more represented in Cocconi. This trend may be related to a transient expansion and altitudinal downward migration of alpine species during the Little Ice Age (approx. datable at the period 1550–1850 [74]). This could be the case of *Geranium argenteum* L., *Juncus trifidus* L. or *Ranunculus montanus* L., which Cocconi even found at less than 400 m.a.s.l. [53]. In Europe, in fact, average summer temperature was 2–3°C lower than in the twentieth century and climate was more arid [75], causing a downward shift of the tree line up to 200 m [76]. This view is supported by the analysis of the geographical ranges of the species, indicating that microthermal plants were more represented in Cocconi than in Aldrovandi's and current floras. This suggests that Cocconi described a flora of the Little Ice Age, whereas Aldrovandi and current data reflect totally different climatic scenarios. The Renaissance marked the end of the Middle Ages climatic *optimum* (e.g. [74]), while currently climate is markedly warming (e.g. [77]).

**Figure 8.**
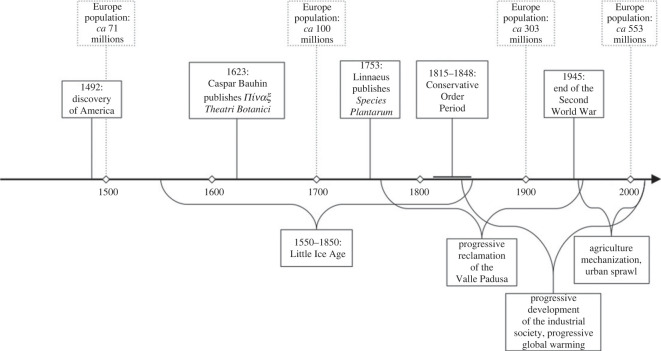
Timeline infography indicating the main historical events occurred in the last five centuries, the principal drivers of landscape and land use change, and the demographic trend of European population (for the latter, that does not take into account the European part of the former USSR, cf. [58,59]).

Our findings also indicate the increasing importance of alien species from the Renaissance to the present day. Aldrovandi's flora refers to a period in which alien species invasion, as currently circumscribed, did not exist and the exotics mostly came from Eurasia, the Palaeotropics and the Mediterranean basin. However, the first signs of a phenomenon that would become irrepressible in the following centuries were already detectable. All the American species currently invading Europe, imported for their ornamental or economic interest (*Acer negundo* L., *Amorpha fruticosa* L., *Robinia pseudoacacia* L. etc.), were not present in Europe at Aldrovandi's time and were naturalized only in very few sites by the end of the nineteenth century [53]. In this perspective, Aldrovandi's herbarium preserves the memory of the first signs of a radical transformation of the European flora and habitats (see also figure 8).

## 5. Conclusion

Besides the demonstration of strong floristic changes across a 500-year timespan, plausibly reflecting variations of climate and land use, our study warns about the risk of ceasing to collect herbarium specimens and keep herbaria active [78,79]. This would cause irreparable gaps to our botanical memory, hindering our ability to document biodiversity and predicting its trajectories just during decades of fast global changes. In this perspective, Aldrovandi's farsighted view of nature should still inspire botanists, stimulating continuous specimen collection as an irreplaceable source of primary data.

## Data Availability

The Erbario Aldrovandi is preserved at the Erbario dell'Università di Bologna (BOLO) and is also available online (http://botanica.sma.unibo.it/); the *Flora della Provincia di Bologna* by Girolamo Cocconi is preserved in some copies in the Biblioteca Storica A. Bertoloni of the Dipartimento di Scienze Biologiche, Geologiche e Ambientali—Università di Bologna and is also available on Google Books; today's floristic data are stored in the Floristic Database of Emilia-Romagna (https://bbcc.ibc.regione.emilia-romagna.it/flora-emilia-romagna/). The data are provided in electronic supplementary material [80].
